# Adaptive Autoimmunity and Foxp3-Based Immunoregulation in Zebrafish

**DOI:** 10.1371/journal.pone.0009478

**Published:** 2010-03-05

**Authors:** Francisco J. Quintana, Antonio H. Iglesias, Mauricio F. Farez, Mario Caccamo, Evan J. Burns, Nasim Kassam, Mohamed Oukka, Howard L. Weiner

**Affiliations:** 1 Center for Neurologic Diseases, Brigham and Women's Hospital, Harvard Medical School, Boston, Massachusetts, United States of America; 2 EMBL Outstation - Hinxton, European Bioinformatics Institute, Wellcome Trust Genome Campus, Hinxton, Cambridge, United Kingdom; 3 Center for Neurologic Diseases, Brigham and Women's Hospital, Harvard Medical School, Cambridge, Massachusetts, United States of America; New York University, United States of America

## Abstract

**Background:**

Jawed vertebrates generate their immune-receptor repertoire by a recombinatorial mechanism that has the potential to produce harmful autoreactive lymphocytes. In mammals, peripheral tolerance to self-antigens is enforced by Foxp3^+^ regulatory T cells. Recombinatorial mechanisms also operate in teleosts, but active immunoregulation is thought to be a late incorporation to the vertebrate lineage.

**Methods/Principal Findings:**

Here we report the characterization of adaptive autoimmunity and Foxp3-based immunoregulation in the zebrafish. We found that zebrafish immunization with an homogenate of zebrafish central nervous system (zCNS) triggered CNS inflammation and specific antibodies. We cloned the zebrafish ortholog for mammalian Foxp3 (zFoxp3) which induced a regulatory phenotype on mouse T cells and controlled IL-17 production in zebrafish embryos.

**Conclusions/Significance:**

Our findings demonstrate the acquisition of active mechanisms of self-tolerance early in vertebrate evolution, suggesting that active regulatory mechanisms accompany the development of the molecular potential for adaptive autoimmunity. Moreover, they identify the zebrafish as a tool to study the molecular pathways controlling adaptive immunity.

## Introduction

The vertebrate immune system uses complex recombinatorial mechanisms to generate a diverse immune receptor repertoire [1]. In jawed vertebrates (gnathostomes), immune repertoire diversity is increased by the incorporation of random mutations in immune receptor genes [2]. This stochastic process can generate autoreactive receptors [1], thus several mechanisms of immunoregulation are in place to prevent the development of autoimmune diseases [3], [4] [5] [6] [7]. For example, in higher gnathostomes like mammals the transcription factor Foxp3 controls the differentiation and function of regulatory T cells (T_reg_) specialized in enforcing self-tolerance in the mature immune system [8], [9], [10]. The lack of functional Foxp3, or even the attenuation of its expression levels results in the development of autoimmune pathology in mice, and has been linked to the autoimmune syndrome immune dysregulation, polyendocrinopathy, enteropathy X-linked (IPEX) in humans [11], [12], [13], [14], [15], [16]. These observations emphasize the importance of Foxp3-driven T_reg_ for the control of the immune response to self- antigens.

The immune system in teleosts like the zebrafish (*Danio rerio*) resembles in several aspects the mammalian immune system. Macrophages, T cells, B cells have been described in teleosts [17], as well as the cytokines IL-17 [18], IFNγ [19] and TNFα [20] and the transcription factors T-bet [21] and retinoid-related orphan receptor [22] which have been linked to mammalian autoimmune pathology. Like in mammals, the immune repertoire of teleosts is generated by recombinatorial and mutational mechanisms [1], [17], [23], [24], [25]. However, although these processes can generate potentially harmful self-reactive immune receptors, the potential for adaptive autoimmunity and mechanisms of immunoregulation have not yet been characterized in lower gnathostomes. Indeed, Foxp3-driven peripheral tolerance has been postulated to be a recent adaptation in vertebrate evolution [23].

Here we have characterized the potential for adaptive autoimmunity and Foxp3-based immunoregulation in the zebrafish. Our results suggest that active mechanisms of peripheral tolerance were incorporated early in vertebrate evolution, together with the development of the molecular potential for adaptive autoimmunity. In addition, considering the experimental advantages offered by the zebrafish as an experimental system [26], [27], our results support the use of the zebrafish for the study of molecular pathways that control immunoregulation.

## Results

### Adaptive Autoimmunity in Zebrafish

**Figure 1:**
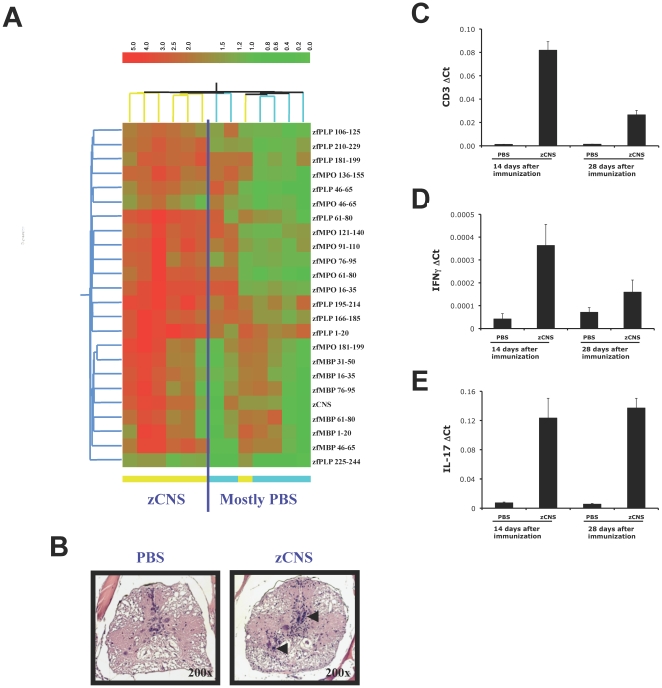
Adaptive autoimmunity in zebrafish. (**A**) Heatmap depicting the autoantibody response to myelin antigens on day 28 after immunization with zCNS or PBS in CFA. Each column represents a serum sample, color-coded at the bottom to indicate whether it corresponds to a zCNS or a control immunized sample. Only significantly up-regulated antibody reactivities are shown (*n* = 8, *t*-test FDR <0.05), according to the colorimetric scale on the right. (**B–E**) Zebrafish were immunized with zCNS or PBS in CFA and 14 or 28 days later the expression of CD3, IL-17 and IFNγ in brain was measured by real time PCR (mean + s.d. of triplicates) (**B–D**) or analyzed histologically for the presence of cell infiltrates (**E**). Two independent experiments produced similar results.

We analyzed the autoimmune response of 6-month old zebrafish immunized intraperitoneally (ip) with zebrafish brain homogenate (zCNS) emulsified in complete Freund's adjuvant (CFA). To study the induction of autoantibodies to zCNS antigens we used zebrafish myelin microarrays [28], [29] in combination with monoclonal antibodies generated against zebrafish immunoglobulins. The zebrafish myelin microarrays contained zCNS and a collection of 39 peptides spanning the whole sequence of zebrafish myelin basic protein (zMBP), zebrafish myelin protein P0 (zMP0) and zebrafish proteolipid protein (zPLP). zCNS immunization induced autoantibodies directed against zCNS and its derived peptides (**Figure 1A**). Moreover, we detected autoimmune encephalomyelitis – the accumulation of CD3, IFNγ and IL-17 expressing cells in the brain of zCNS-immunized zebrafish (**Figures 1B–E**). Thus zebrafish can mount adaptive antigen-specific autoimmune responses.

### Zebrafish Foxp3 (zFoxp3)

**Figure 2:**
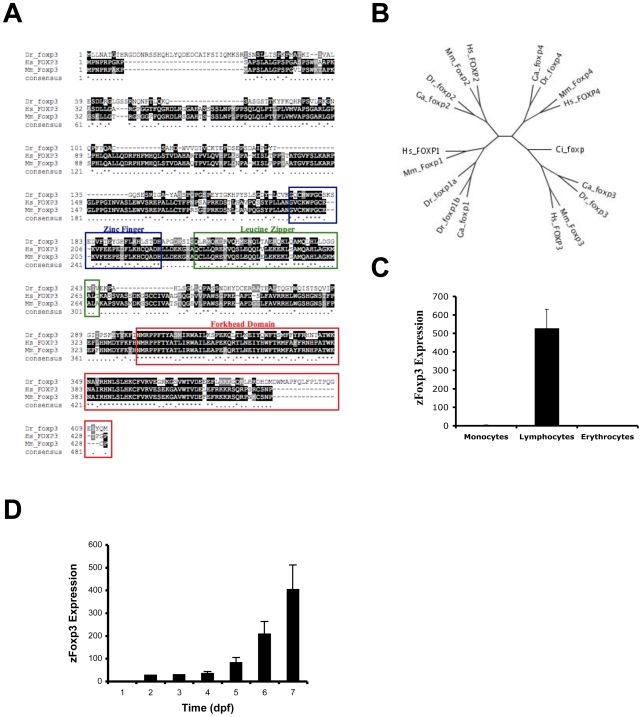
Zebrafish Foxp3 (zFoxp3). (**A**) Sequence comparison of putative FoxP3 genes of zebrafish, human and mouse. The stars indicate identity, dashes were introduced for optimal alignment. The zinc finger, leucine zipper and forkhead domains are highlighted with a blue, green or red box, respectively. (**B**) Radial gene tree showing the Foxp1, Foxp2, Foxp3 and Foxp4 proteins in mammals and fish, where the *Ciona intestinalis* Foxp sequence is the outgroup. The branch lengths are proportional to the distance between the sequences. Mm, *Mus musculus*; Hs, *Homo sapiens*; Dr, *Danio rerio*; Ga, *Gasterosteus aculeatus* (stickleback); Ci, *Ciona intestinalis*. The accession numbers for the amino acid sequences used in the gene tree analysis are as follows: *Danio rerio* Foxp1a Q08BX8 BC124513; Foxp1b Q2LE08 NM_001039637; Foxp2 Q4JNX5 NM_001030082; Foxp3 annotated (EST CK028390); Foxp4 annotated. *Homo sapiens:* Foxp1 Q9H334 NM_001012505, Foxp2 O15409 NM_148899, Foxp3 Q9BZS1 NM_014009, Foxp4 Q8IVH2 NM_138457; *Mus musculus*: Foxp1 P58462 NM_053202, Foxp2 P58463 NM_053242, Foxp3 Q99JB6 NM_054039, Foxp4 Q9DBY0 NM_028767; *Ciona intestinalis* Foxp Q4H3H6. The amino acid sequence of the apparent stickleback orthologues of Foxp1, Foxp2, Foxp3 and Foxp4 were obtained from Ensembl. (**C**) Monocytes, lymphocytes and erythrocytes were sorted by FACS and the expression of zFoxP3 was determined by real time PCR (mean + s.d. of triplicates). (**D**) zFoxp3 and GAPDH were quantified by qPCR on cDNA prepared from zebrafish embryos at different times after fertilization. Two independent experiments produced similar results.

The potential to mount adaptive antigen-specific autoimmune responses suggested that zebrafish harbor mechanisms that limit the activity of the immune system. Several mechanisms have been described to control the immune response in vertebrates, one of those mechanisms is the suppressor activity of Foxp3-driven T_reg_ [8], [9], [10]. To study whether FoxP3-dependent immunoregulation exists in zebrafish, we searched the zebrafish genomic sequence for a FoxP3 homologue. We found a zebrafish homologue for mammalian Foxp3 and termed it zFoxp3 (**Figure 2A**). Phylogenetic analysis placed zFoxp3 in a sub-tree together with mammalian and other fish orthologous predictions, suggesting that zFoxp3 is the zebrafish ortholog for mammalian Foxp3 (**Figure 2B**). Foxp3 in mammals is located in a well-conserved synteny block. Indeed, we found several orthologous genes between mammalian chromosome X and the region surrounding the *zfoxp3* locus in zebrafish chromosome 8 (suv39h1, cacna1s, tspyl2, wasp), strengthening the likehood of zFoxp3 being the fish ortholog of Foxp3. Western blot studies of zebrafish tissues identified a band of a molecular weight compatible with the predicted size of zFoxp3 cross-reactive with Foxp3 (data not shown). We confirmed our western blot results by studying the expression pattern of zFoxp3 by real-time PCR on FACS sorted lymphocytes, myelomonocytes and erythrocytes [30]: zFoxp3 expression was restricted to the lymphocyte fraction (**Figure 2C**). A longitudinal follow up in developing embryos revealed zFoxp3-detectable expression in 5–6 day post-fertilization embryos (**Figure 2D**).

### zFoxp3 Is a Functional Homologue of Mammalian Foxp3

**Figure 3:**
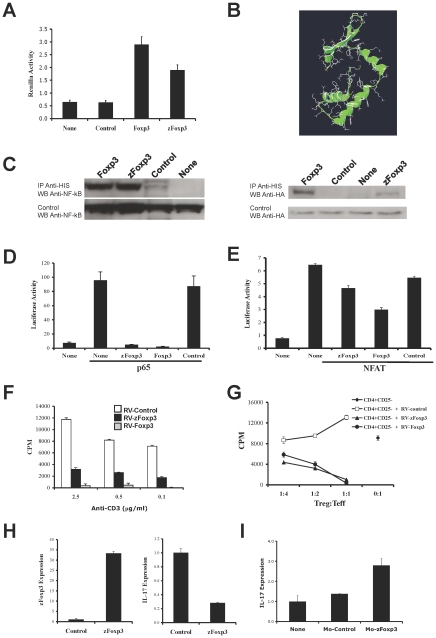
zFoxp3 is a functional homologue of mammalian Foxp3. (**A**) Constructs coding for His-labeled zFoxp3 and Renilla-labeled Foxp3 were co-transfected into 293T cells. 24 h later the cells were lysed, zFoxp3 was pulled-down with Ni-Agarose and the renilla luciferase activity in the pellet was quantified. The results are normalized for the total amount of luciferase before precipitation (mean + s.d. of triplicates). Three independent experiments produced similar results. (**B**) Structure of the forkhead domain of zFoxp3 obtained by homology modeling, based on the structure of the crystallized forkhead domain of Foxp1. (**C**) 293T cells were co-transfected with His-tagged zFoxp3, Foxp3 and NF-kB or HA-flagged NFAT and 24 hr later the cells were lyzed and immunoprecipitated with antibodies to His antibodies. The precipitates were resolved by PAGE-SDS and detected by western blot with antibodies to NF-kB or HA antibodies. Three independent experiments produced similar results. (**D, E**) 293T cells were co-transfected with reporter constructs coding for luciferase under the control of a NF-kB or NFAT responsive promoters, and p65 NF-kB or NFAT in the presence of vectors coding for zFoxp3, Foxp3 or control (empty vector). Luciferase activity was normalized to the renilla activity of a co-transfected control (mean + s.d. of triplicates). Four independent experiments produced similar results. (**F**) MACS-purified CD4^+^CD25^−^ T-cells were transduced with a bicistronic retrovirus coding for GFP and zFoxp3, Foxp3 or an empty control retrovirus, and the GFP^+^ population was analyzed for its proliferation upon activation with plate bound antibodies to CD3 (mean cpm or pg/ml + s.d. in triplicate wells) and (**G**) its suppressive activity on the proliferation and IL-2 and IFNγ secretion of mouse CD4^+^CD25^−^ T-cells activated with plate-bound antibodies to CD3 (mean cpm or pg/ml + s.d. in triplicate wells). Two independent experiments produced similar results. (**H**) Fertilized zebrafish eggs were microinjected with a plasmid coding for zFoxp3 or an empty plasmid, and zFoxp3 and IL-17 expression were measured from 6 days old embryos by real time PCR. Two independent experiments produced similar results. (**I**) Fertilized zebrafish eggs were microinjected with a morpholino oligonucleotides designed to interfere with the translation of zFoxP3 (Mo-zFoxp3) or a 5 bases mismatch control oligonucleotide and IL-17 expression was measured in 5 days old embryos by real time PCR. Two independent experiments produced similar results.

Mammalian Foxp3 has to dimerize to be transcriptionally active [31]. To evaluate the dimerization capability of zFoxp3, we designed a pull-down assay in which we co-transfected a plasmid coding for a His-tagged zFoxp3 with a construct coding for Foxp3 fused to Renilla luciferase (Ren). After 24 hr, the cells were lysed, precipitated with Ni-Agarose and Ren activity was measured in the pellet. **Figure 3A** shows that zFoxp3 can homodimerize and pull-down Foxp3-Ren.

The forkhead domain in FOX proteins mediates their interactions with DNA and with other transcription factors [10], [32]. The amino acids (aa) that mediate the interaction with the DNA in mammalian Foxp3, as well as aa targeted by inactivating mutations in humans with impaired Foxp3 activity [10], [32], [33], were found to be conserved in zFoxp3 (**Figure 2A**). Indeed, protein-structure homology modeling of the forkhead domain in zFoxp3 indicated that it displays the characteristic winged-helix structure described in FOXP proteins [33] (**Figure 2B**).

Foxp3 interacts with NF-kB and NFAT to inhibit their transcriptional activities [32], [34]. We found that the aa that mediate the interaction with NFAT [32] are conserved in zFoxp3 (**Figure 2A**). Moreover, his-tagged zFoxp3 co-precipitated with NF-kB and NFAT, although the interaction with NFAT seems to be weaker (**Figure 3C**). Co-transfection experiments with NF-kB and NFAT responsive reporters revealed that zFoxp3 interfered with the activity of NFAT and NF-kB responsive promoters (**Figure 3D–E**). In agreement with our coprecipitation results, zFoxp3 showed reduced inhibitory effects on NFAT-driven reporters (**Figure 3E**). Thus zFoxp3 displays structural characteristics similar to those of mammalian Foxp3 that allow it to interact with NFAT and NF-kB and interfere with their transcriptional activities.

T_reg_ cells are characterized by a decreased proliferative and cytokine response to activation, and this phenotype can be recapitulated by over-expression of FOXP3 in naïve T cells [9]. Viral transduction of *z*Foxp3 into murine T cells resulted in a significant decrease in proliferation (**Figure 3F**). Moreover, zFoxp3 transduced T cells could suppress the activation of other T cells in co-culture assays (**Figure 3G**).

To further investigate the function of zFoxp3 in zebrafish, we over-expressed zFoxp3 in zebrafish developing embryos. Microinjection with zFoxp3-expressing constructs resulted in an up-regulation of zFoxp3 levels, concomitant with the down-regulation of IL-17 levels (**Figure 3H**). Conversely, microinjection with morpholino oligonucleotides designed to block the translation of zFoxp3 led to the upregulation of IL-17 expression, which was not observed upon the injection of 5 bases mismatch negative control morpholino (**Figure 3I**). All in all, these results indicate that *z*Foxp3 is the functional homologue of Foxp3 in mammals.

### AHR Controls zFoxp3 Expression

**Figure 4:**
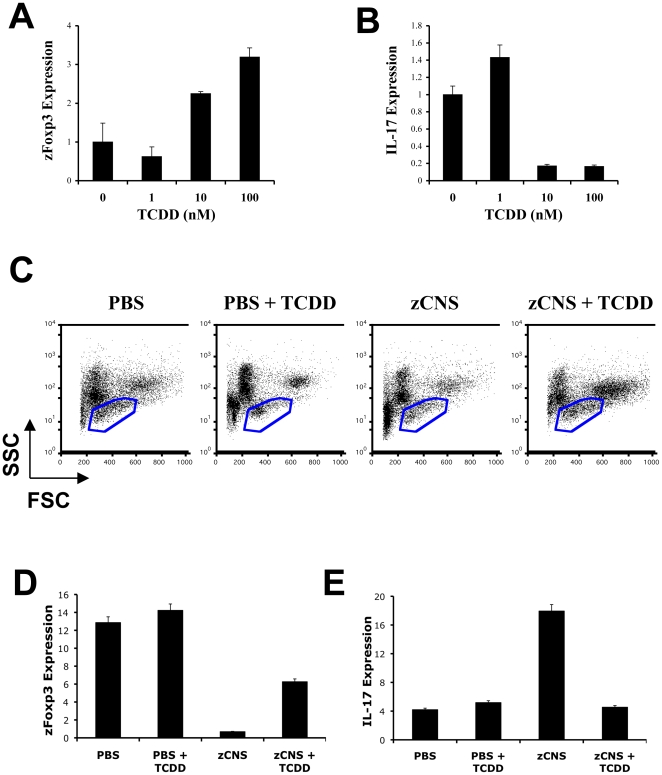
AHR controls zFoxp3 expression. (**A,B**) TCDD was added to the water of three-day post-fertilization zebrafish embryos, and 72 h later zFoxp3 (**A**) and IL-17 (**B**) expression were determined by real time PCR (mean + s.d. of triplicates normalized to GAPDH expression). Two independent experiments produced similar results. (**C**) Fourteen days after immunization, kidney cells from PBS or zCNS immunized zebrafish, or TCDD-treated zCNS immunized zebrafish were analyzed by FACS and cells in the lymphocyte fraction (blue gate) were sorted. (**D–E**) Expression of zFoxp3 and IL-17 measured by real-time PCR in FACS-sorted lymphocytes. Two independent experiments produced similar results.

We have recently reported that the ligand-activated transcription factor aryl hydrocarbon receptor (AHR) controls the expression of mouse Foxp3 [28]. Indeed, it has been recently reported that AHR is expressed by lamprey lymphocytes that resemble T cells [35]. The restricted expression of zFoxp3 on zebrafish lymphocytes suggested that at least some of the signaling pathways controlling Foxp3 expression are evolutionarily conserved (**Figure 2C–D**). To test this hypothesis we analyzed the effect of AHR activation by its high affinity ligand 2,3,7,8-tetrachlorodibenzo-p-dioxin (TCDD) on zFoxp3 expression. The treatment of developing zebrafish embryos with TCDD resulted in a dose-dependent increase in zFoxp3 expression (**Figure 4A**); this increase was concomitant with a down regulation in IL-17 expression (**Figure 4B**). Moreover, treatment of zCNS immunized fish with TCDD resulted in an increase in Foxp3 expression in the lymphocyte fraction, and a concomitant decrease in the expression of IL-17 (**Figure 4C–E**). These results suggest that zebrafish AHR controls Foxp3 and T_reg_, as shown in the mouse [28], [36].

## Discussion

Cells with the morphological and functional features of lymphocytes are first found in agnathans (jawless vertebrates) of which the only two non-extinct representatives are the lamprey and the hagfish [25]. The paleontological record suggests that agnathans are basal to gnathostomes [37], thus lymphocytes are thought to have evolved in the most basal vertebrates or protochordate ancestor [1], [24], [25]. Agnathans and gnathostomes, however, differ both in the nature of their lymphocytes and in the strategies used for the generation of their immune receptors. Agnathans' lymphocytes resemble B cells [24], [38] which can be engaged in T-independent type immune responses [39]. Their immune receptors achieve a diversity similar to that of mammalian antibodies by combining preexisting receptor blocks through a mechanism of gene conversion [38]. Consequently, agnathans are thought to generate a self-tolerant immune repertoire through the evolutionary selection for non-autoreactive receptor building blocks [23].

T cells, lymphocytes bearing variable immune receptors that interact with target peptides bound to major histocompatibility complex molecules are only found in jawed vertebrates [1], [23], [24], [25], [40]. Gnathostomes combine immune-receptor building blocks through a process that incorporates stochastic mutations and has therefore the potential to generate autoreactive receptors [1], [23]. Mammalian T cells are purged from self-reactivity at the thymus [4], and similar mechanisms are thought to remove autoreactive T cells in the zebrafish thymus [17], [41]. The thymic deletion of autoreactivity, however, is incomplete [4]. Indeed some degree of self reactivity is required to complete the thymic maturation of nascent T cells [4] and ensure the survival [42] and activity [43] of the mature T cells in the periphery. This self-reactivity, unless regulated, can result in autoimmune pathology [6].

**Figure 5:**
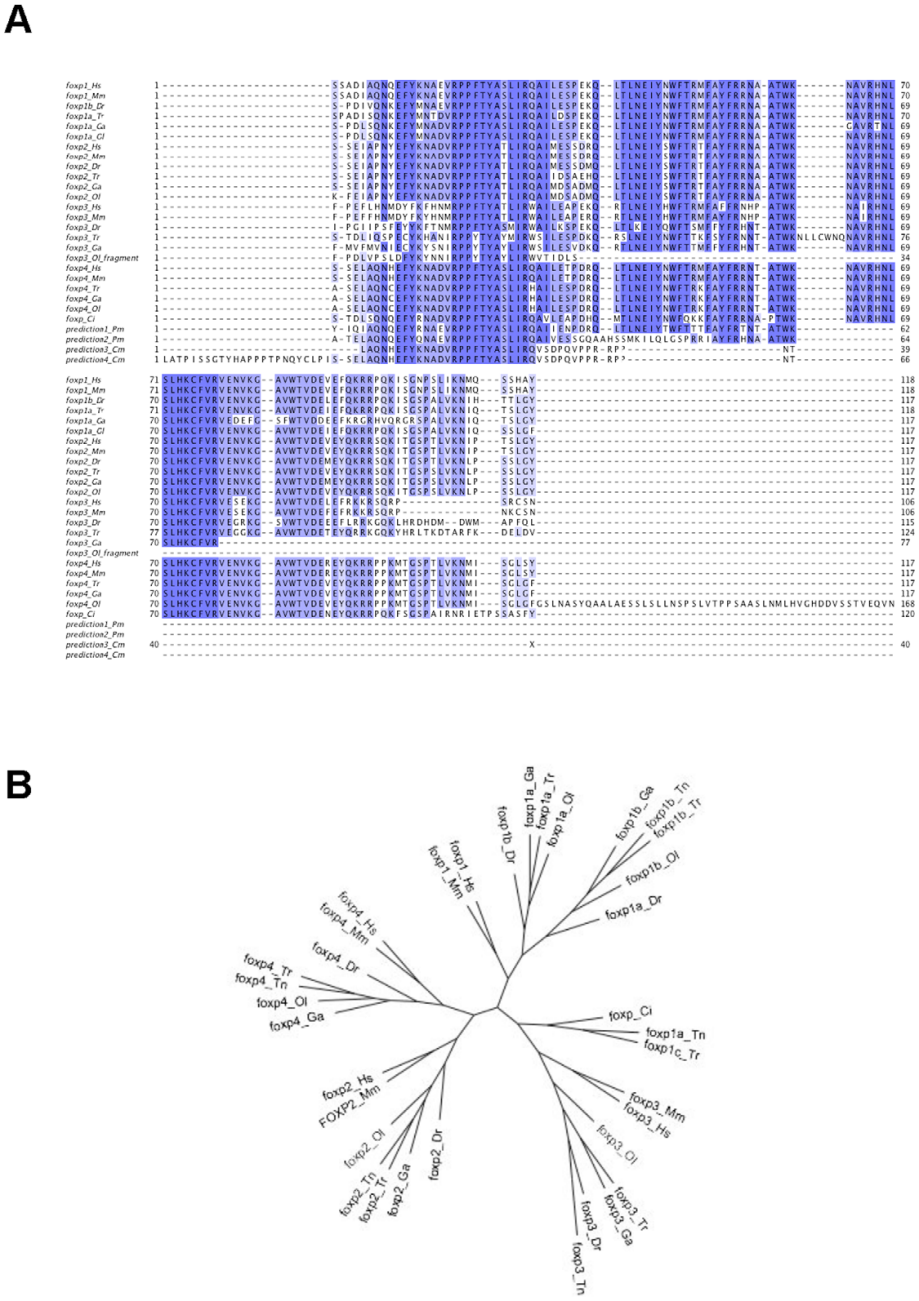
Evolutionary conservation of the forkhead domains of the FOX family. (**A**) Multiple sequence alignment for the forkhead domains of the FOX family. The alignment includes the sequence for Foxp1–4 for *Homo sapiens*, *Mus musculus* (Genbank/EMBL sequences) and several of the sequences for the teleosts *Danio rerio* (Dr), *Oryzias latipes* (Ol), *Takifugu rubripes* (Tr) and *Gasterosteus aculeatus* (Ga) (Ensembl annotation). The partial sequences prediction1_Pm and prediction2_Pm were obtained by assembling sequenced sea lamprey reads. Similarly the sequences prediction3_Cm ad prediction4_Cm are from sequenced reads from the elephant shark genome project (*Callorhinchus milii*). Although these predicted sequences show characteristic FOXP motifs, as for example the N-terminus motif RPPFTYA, there is strong indication that they are not part of FOXP3 forkhead domains, as for example the motif LIAQAI that in FOXP3 forkhead is replaced by LIRWAI. (**B**) Radial gene tree for the forkhead domains of the FOX family showing the Foxp1, Foxp2, Foxp3 and Foxp4 proteins in mammals and fish, where the *Ciona intestinalis* Foxp sequence is the outgroup. The branch lengths are proportional to the distance between the sequences.

Mammals control self-reactivity by various mechanisms including the activity of Foxp3-driven T_reg_ [8], [9], [10]. Our results on zebrafish Foxp3 point to the early acquisition of T_reg_-based tolerance during vertebrate evolution, together with thymic mechanisms of positive and negative selection. A functional thymus cannot be identified in the lamprey[44], [45]. Accordingly, our efforts to identify *foxp3* in the sequenced reads of the lamprey genome available in public repositories (estimated to represent an 8x coverage) have been so far unsuccessful. We have not found a *foxp3* ortholog in cartilaginous fish, but the low coverage (2X) of the elephant shark (*C. milii*) genome and the restricted number of available EST prevents us from making any definitive conclusion about *foxp3* in this group. We could identify, though, coding regions consistent with *C. intestinalis* and purple sea urchin *foxp* genes, but the analysis of characteristic sequence motifs indicates that these genes are not *foxp3* orthologous (**Figure 5**). Thus, we propose that mechanisms of central (thymic) and peripheral (T_reg_) tolerance were co-selected early during vertebrate evolution, together with T cell-like lymphocytes. The completion of the lamprey genome project, and the sequencing of the genomes of cartilaginous fish and other vertebrates such as the hagfish might lend support to this interpretation.

*C. elegans* and *D. melanogaster* have been extremely useful for the identification of the genes governing innate immunity [46]. These experimental models, however, lack an adaptive immune system, limiting their use to study immunoregulation. Our data suggest an evolutionary conservation of mechanisms and signaling pathways that control adaptive immunity in mammals and zebrafish. Based on the advantages that it offers for the realization of genetic and chemical screens [26], [27], the zebrafish might constitute a new tool for the identification of targets and drug candidates for autoimmunity.

## Materials and Methods

### Cloning of zFoxp3

zFoxp3 was cloned from cDNA prepared from zebrafish kidney by using a TOPO® PCR cloning kit (Invitrogen, CA, USA) according to the manufacturer's instructions.

### Mice

C57BL/6 mice were purchased from Jackson Laboratories (Bar Harbor, Maine, USA). Mice were kept in a conventional, pathogen-free facility at the Harvard Institutes of Medicine. All experiments were carried out in accordance with guidelines prescribed by the Institutional Animal Care and Use Committee (IACUC) at Harvard Medical School.

### Zebrafish Immunization

Six month old zebrafish were anesthetized with 0.02% tricaine (Sigma-Aldrich) and immunized ip with 5 µl/fish of antigen emulsified in CFA.

### FACS Sorting

Adult wild-type adult zebrafish were anaesthetized with 0.02% tricaine and the spleen and kidney were dissected and placed in PBS as described. Single cell suspensions were generated by passing through a nylon mesh and stained with propidium iodide (Sigma) was to exclude dead cells, and sorted based on propidium iodide exclusion, forward scatter and side scatter as described [30], using a FACSVantage flow cytometer (Beckton Dickinson) at the *FACS* Core Facility at Children's Hospital, Boston.

### Real time PCR

RNA was extracted from cells using RNAeasy columns (Qiagen, Valencia, CA, USA), complementary DNA was prepared as recommended (Bio-Rad Laboratories, Hercules, CA, USA) and used as template for real time PCR. The expression of Foxp3 was quantified with specific primers and probes (Applied Biosystems, Foster City, CA, USA) on the GeneAmp 5500 Sequence Detection System (Applied Biosystems). Expression was normalized to the expression of the housekeeping gene, GAPDH.

### Transfection, Luciferase and Immunoprecipitation Assays

293 cells were transfected as described [34] and the cells were analyzed after 24 or 48 h with the dual luciferase assay kit (New England Biolabs, Ipswich, MA). Tk-Renilla was used for standardization. Alternatively, the transfected cells were lysed and immunoprecipitation was carried out as described [34]; hemagglutinin (HA) labeled NFAT and NF-kB were detected with anti-HA and anti-P65 antibodies obtained from Santa Cruz Biotechnology (Santa Cruz, CA, USA), respectively.

### Retroviral Infection

MSCV GFP-RV retroviral DNA plasmids were transfected into the Phoenix packaging cell line and 72 h later the retrovirus-containing supernatants were collected. MACS-purified CD4^+^ T cells were activated 24 h with plate-bound antibodies to CD3 and CD28, and infected by centrifugation (45 min at 2000 rpm) with retrovirus-containing supernatant supplemented with 8 µg/ml Polybrene (Sigma-Aldrich) and recombinant human IL-2 (25 units/ml).

### Cell Proliferation

Cells were cultured in serum-free X-VIVO 20™ media (BioWhittaker) for 72 h. During the last 16 h, cells were pulsed with 1 muCi of [3H]thymidine (PerkinElmer) followed by harvesting on glass fiber filters and analysis of incorporated [3H]thymidine in a beta-counter (1450 Microbeta, Trilux, PerkinElmer).

### Antigen Arrays

The antigens listed in **Table S1** were spotted onto Epoxy slides (TeleChem, Sunnyvale, CA, USA) as described [29]. Antigens were spotted in replicates of 6, the microarrays were blocked for 1 h at 37°C with 1% bovine serum albumin, and incubated for 2 h at 37°C with a 1∶100 dilution of the test serum in blocking buffer. The arrays were then washed and incubated for 45 min at 37°C with mouse anti-zebrafish Ig and for 45 min at 37°C with goat anti-mouse IgG Cy3-conjugated detection antibodies (Jackson ImmunoResearch Labs, West Grove, PA, USA). The arrays were scanned with a ScanArray 4000X scanner (GSI Luminomics, Billerica, Massachusetts, USA). Antigen reactivity was defined by the mean intensity of binding to the replicates of that antigen on the microarray. Raw data were normalized and analyzed using the GeneSpring software (Silicon Genetics, Redwood City, CA, USA) with the non-parametric Wilcoxon-Mann-Whitney test, using the Benjamini and Hochberg method with a false discovery rate (FDR) of 0.05 to determine significance [47]. The samples were clustered using a pairwise average linkage algorithm based on Spearman's rank correlation as a distance measure [47].

### Monoclonal Antibodies to Zebrafish Immunoglobulins

We synthesized three peptides, each one corresponding to a different domain in the heavy chain of zebrafish IgM, and conjugated them to BSA or KLH through a C-terminal cysteine using appropriate kits (Pierce Biotechnology Inc., Rockford, IL 61105, US). The sequence of the peptides used are: M1, LNFKWKDPAGKDLSDFVQYPC; M2, TASLAPPAPPPDLRATVFLTC; M3, AVDNFFKDEKNGSVTEYSATC. In spleen extracts of adult zebrafish, monoclonal antibodies generated against these peptides recognized proteins of a molecular weight compatible with IgM heavy chain were (data not shown).

### Zebrafish Microinjection

Zebrafish eggs were collected within 1 hr of spawning, and purified plasmids or morpholino antisense oligonucleotides were microinjected with a fine glass needle connected to an automatic injector. A morpholino oligonucleotide designed to block the translation of zFoxp3 (5′-GTGTTCCAGTAGCATTAAGAAGCAT-3′) and a 5 bases mismatch control oligonucleotide (5′-GTcTTCgAGTAcCATTAAcAAGgAT-3′) were designed and synthesized by Gene Tools (Philomath, OR). Each morpholino nucleotide was injected into the yolk of embryos at one to four cell stages.

## Supporting Information

Table S1 Antigens used to construct antigen microarrays
(0.02 MB XLS)
